# Induction of colorectal carcinogenesis in the C57BL/6J and A/J mouse strains with a reduced DSS dose in the AOM/DSS model

**DOI:** 10.1186/s42826-021-00096-y

**Published:** 2021-07-27

**Authors:** Henriette Arnesen, Mette Helen Bjørge Müller, Mona Aleksandersen, Gunn Charlotte Østby, Harald Carlsen, Jan Erik Paulsen, Preben Boysen

**Affiliations:** 1 Department of Preclinical Sciences and Pathology, Faculty of Veterinary Medicine, Norwegian University of Life Sciences (NMBU), Oslo, Norway; 2 Faculty of Chemistry, Biotechnology and Food Science, Norwegian University of Life Sciences (NMBU), Ås, Norway; 3 Department of Paraclinical Sciences, Faculty of Veterinary Medicine, Norwegian University of Life Sciences (NMBU), Oslo, Norway; 4 Department of Production Animal Clinical Sciences, Faculty of Veterinary Medicine, Norwegian University of Life Sciences (NMBU), Oslo, Norway

**Keywords:** AOM/DSS, Colorectal cancer, Mouse models, Disease models, Azoxymethane, Dextran sulfate sodium

## Abstract

**Background:**

Colorectal cancer (CRC) is one of the most frequently diagnosed cancers worldwide and thus mouse models of CRC are of significant value to study the pathogenesis. The Azoxymethane/Dextran sulfate sodium (AOM/DSS) model is a widely used, robust initiation-promotion model for chemical induction of colitis-associated CRC in rodents. However, the dosage of chemicals, treatment regimens and outcome measures vary greatly among studies employing this model. Thus, the aim of this study was to examine an AOM/DSS model involving a reduced (1%) dose of DSS for induction of carcinogenesis in A/J and C57BL/6J (B6) mice.

**Results:**

We show that colonic preneoplastic lesions can be reliably detected in A/J and B6 mice by use of a AOM/DSS model involving a single injection of 10 mg/kg AOM followed by three 7-day cycles of a low-dose (1%) DSS administration. Supporting existing evidence of A/J mice exhibiting higher susceptibility to AOM than B6 mice, our AOM/DSS-treated A/J mice developed the highest number of large colonic lesions. Clinical symptoms in both strains subjected to the AOM/DSS treatment did not persist in-between treatment cycles, demonstrating that the animals tolerated the treatment well.

**Conclusions:**

Our findings suggest that a reduced dose of DSS in the AOM/DSS model can be considered in future studies of early phase colorectal carcinogenesis in the A/J and B6 mouse strains using preneoplastic lesions as an outcome measure, and that such regimen may reduce the risk of early trial terminations to accommodate human endpoints. Overall, our data emphasize the importance of devoting attention towards choice of protocol, outcome measures and mouse strain in studies of CRC in mice according to the study purpose.

**Supplementary Information:**

The online version contains supplementary material available at 10.1186/s42826-021-00096-y.

## Background

Colorectal cancer (CRC) is the second most diagnosed cancer in women, and the third most in men worldwide [1, 2]. CRC etiology is not known, but numerous risk factors have been characterized. A minority of CRC cases are attributed to hereditary factors, such as germline mutations in susceptibility genes, while most CRCs arise sporadically with inflammation being a well-established driver. Individuals with inflammatory bowel diseases (IBDs) such as ulcerative colitis and Crohn’s disease have substantially increased risk of CRC [3, 4]. Generally, the initial changes in colorectal carcinogenesis involve formation of hyperplastic and dysplastic crypts that in turn proliferate to form microadenomas giving rise to adenomatous polyps, carcinoma and ultimately invasive cancer [5–7]. A variety of preneoplastic lesions reported to be involved in the initiation of colorectal tumorigenesis are widely used as biomarkers of colorectal carcinogenesis. The preneoplastic lesions characterized in rodents include aberrant crypt foci (ACF) [8], flat aberrant crypt foci (flat ACF) [9, 10], mucin-depleted foci (MDF) [11] and β-catenin accumulated crypts (BCAC) [12], that may partly overlap [13].

Experimental mouse models that mimic the initiation and progression of CRC is of great importance to study causes, mechanisms and preventive agents. Genetic models such as the Min (multiple intestinal neoplasia) mice harboring a mutant allele of the murine *Apc* (adenomatous polyposis coli) gene are widely used [14]. Another approach is via chemical induction, such as administration of the specific colorectal pro-carcinogen azoxymethane (AOM) [14]. Following metabolism by CYP2E1, AOM is converted to methylazocymethanol (MAM), a highly reactive alkylating species that generate O^6^ mehylguanine adducts in DNA resulting in mutation accumulation and induction of carcinogenesis [14–16]. A model involving a combinatory treatment with AOM and dextran sulfate sodium (DSS) salt, called the AOM/DSS model, was developed to mimic human colitis-associated CRC [17]. DSS is a heparin-like polysaccharide that inflict colonic epithelial damage, mucosal permeability and transmural inflammation in mice [18–20]. The AOM/DSS model is considered robust and reproducible, and it has emerged to become one of the most frequently used models to study inflammation-associated colorectal carcinogenesis in rodents [14, 21–23].

Commonly, AOM/DSS protocols involve one or more AOM injections followed by colitis induced by repeated cycles of 2–3% (w/v) DSS administered via drinking water [14, 17, 22]. Nevertheless, the administered doses of AOM and DSS vary greatly between studies, and there is no consensus practice for this experimental model. Tumorigenic effects of AOM combined with a low dose of DSS (≤1%) has been reported in various mouse strains [24, 25]. Yet, to our knowledge, only a few reports have since employed AOM/DSS models with DSS doses lower than 2% for the study of agents influencing colorectal carcinogenesis in mice [26–29]. Dependent on the doses and frequency of DSS administration as well as experimental timespan, AOM/DSS treatment can lead to consistent development of adenomas and adenocarcinomas [24, 26] or inconsistent tumor incidence [24, 27].

An increasing body of evidence have also emphasized high discrepancies with respect to susceptibility to DSS and AOM in different inbred mouse strains. The A/J mice has been shown to be among the most susceptible strains to AOM-induced colorectal carcinogenesis while C57BL/6J (B6) has been reported relatively resistant [25, 30, 31]. Furthermore, the B6 strain is reportedly more susceptible to DSS-induced inflammation compared to other strains [32]. Based on this knowledge, adjustments of treatment regimen for the susceptibility the mouse strain is recommended prior to design of experiments employing the AOM/DSS model.

Clinical symptoms following AOM/DSS treatments are caused by colitis or advancement of cancer itself, and can include body weight loss, bloody stool and rectal bleeding, rectal prolapse, as well as early death [20, 21, 24, 33]. However, observed symptoms following treatments are often scarcely documented in studies employing AOM/DSS models. Moreover, outcome measures vary between AOM/DSS studies, with count of macroscopically visible tumors and histopathological assessment being the dominant endpoints. The use of preneoplastic lesions such as ACFs as biomarkers has been commended to minimize animal suffering in CRC experiments [34], which is in compliance with the European directive 2010/63 on the protection of animals used for scientific purposes [35].

In this study, we evaluated an AOM/DSS model involving a single injection of AOM in combination with three treatment cycles of a reduced (1%) dose of DSS for induction of carcinogenesis in colons of A/J and B6 mice, reportedly representing two extremes with respect to AOM susceptibility. We hypothesized that ACFs are reliably detected in colons of mice subjected to such treatment approach, and thus enables modelling of the induction phase of carcinogenesis while limiting adverse clinical symptoms.

## Results

### General findings and clinical evaluation

**Fig. 1:**
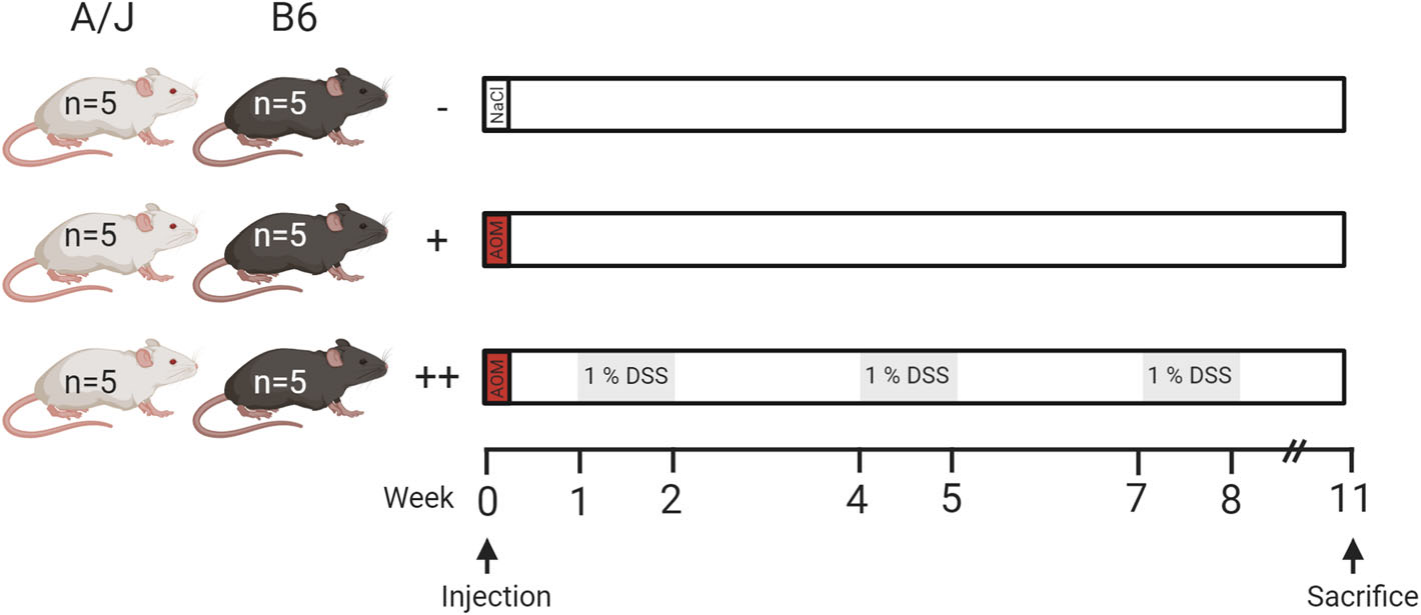
Experimental design. Figure shows grouping and timeline of the experiment. A/J and C57BL/6J (B6) mice were randomized to groups administered NaCl injection (−), AOM-injection (+), or AOM-injection combined with three cycles of 1% DSS treatment (++). All animals were sacrificed at week 12. Figure was created with elements from BioRender.com

**Fig. 2:**
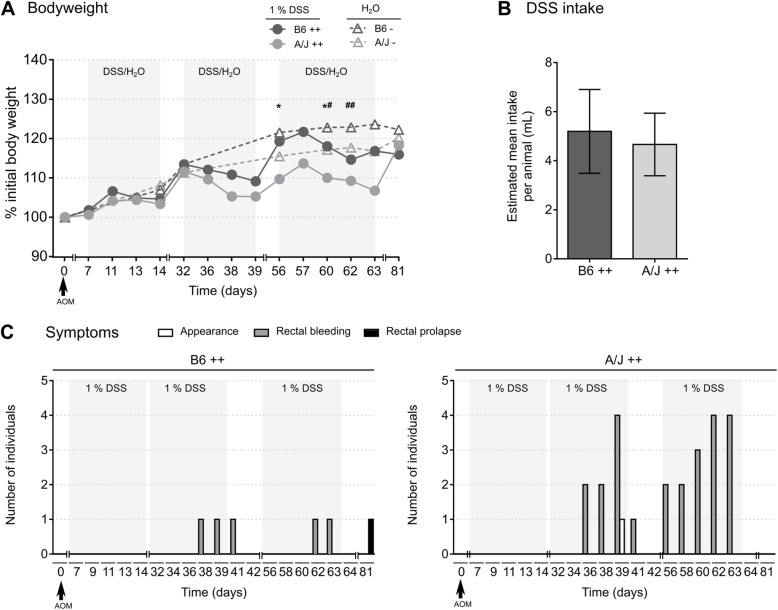
Assessment of body weight, fluid intake and symptoms following AOM/DSS treatment. **(A)** Body weight curves for AOM/DSS (++) and control (−) treated B6 and A/J mice, presented as mean. DSS/H_2_O treatment periods are highlighted with grey background. Statistical differences were determined over the timepoints where data was collected for all groups, using repeated-measures two-way ANOVA with Greenhouse-Geisser correction and Tukey’s multiple comparisons tests (A/J++ vs B6++ *p ≤ 0.05; B6++ vs B6- #*p* ≤ 0.05, ##*p* ≤ 0.01). *n* = 5 in each group. **(B)** DSS intake for AOM/DSS (++) treated B6 and A/J mice. Bars represent estimated average intake per animal, calculated based on drinking bottle volumes for each cage, presented as mean (SD) of mean intake for the whole treatment regimen. **(C)** Number of individuals in the AOM/DSS (++) treated B6 (left) and A/J (right) mice exhibiting clinical symptoms of abnormal appearance, rectal bleeding (including blood in feces) and/or rectal prolapse at different timepoints during the trial. DSS treatment periods are highlighted with grey background

Wild-type A/J mice were administered an AOM injection, an AOM injection combined with DSS treatment, or control treatment (Fig. 1). For the AOM/DSS–treated animals, bodyweight and clinical symptoms were registered every 2nd day throughout the DSS treatment periods. Neither of these groups showed significant reduction in bodyweight loss during the DSS treatment cycles (Fig. 2A). Comparison of the bodyweight change between the AOM/DSS–treated and control groups revealed a significant interaction effect (*p* < 0.001) of group and time. Pairwise comparisons showed a significantly lower bodyweight in the AOM/DSS–treated A/J mice compared to the AOM/DSS–treated B6 mice at day 56 (*p* = 0.048) and 60 (*p* = 0.050), and a significantly lower bodyweight in the AOM/DSS–treated B6 compared to the control B6 group at day 60 (*p* = 0.031) and 62 (*p* = 0.010). Intake of DSS was similar for the B6 and A/J mice, indicating differences in disease states were not biased by inconsistent DSS consumption (Fig. 2B).

Both A/J and B6 strains exhibited clinical symptoms during AOM/DSS treatment, with A/J mice showing the most prominent symptoms. The first symptoms appeared during the 2nd DSS cycle, with observed blood in feces of both A/J mice (day 36) and B6 mice (day 38) (Fig. 2C). Throughout the DSS treatment regimens, blood in feces was observed in all AOM/DSS–treated A/J individuals, while only in two B6 individuals. Rectal bleeding, defined as blood around anus, was observed in two A/J individuals at the end of the 3rd cycle of DSS treatment. Rectal bleeding was also observed in two B6 mice at the end of the 2nd and 3rd cycle of DSS treatment, respectively. All animals did, however, convalesce during the subsequent recovery periods. Appearance and behavior were normal for all animals, except one A/J individual during the 2nd DSS cycle (day 39) that convalesced during the subsequent recovery period. One individual in B6 AOM/DSS group showed signs of rectal prolapse at termination. All AOM and control treated animals appeared healthy throughout the trial, with no recorded symptoms or bodyweight loss (**S1 dataset**).

### Identification of intestinal lesions

**Fig. 3:**
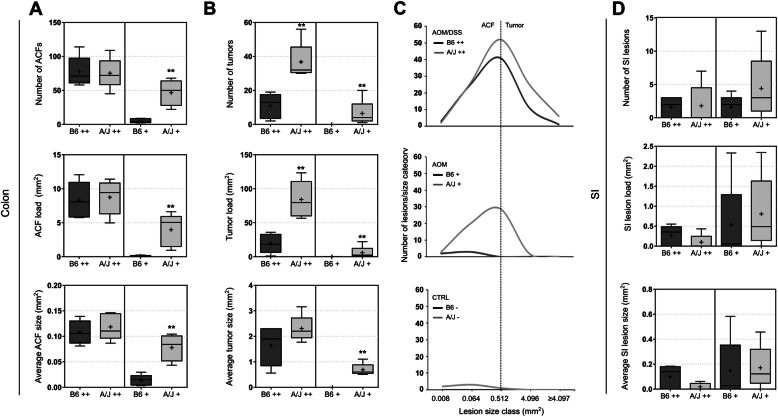
Scoring of intestinal lesions. **(A, B)** Number, load and average size of colonic lesions in B6 or A/J mice treated with AOM/DSS (++) or AOM only (+). Lesions were categorized as either ACFs (≤30 abnormal crypts) or tumors (≥30 abnormal crypts). Box plots show median (line), mean (+), IQR (box) and minimum to maximum (whiskers). Statistical significance between strains treated with AOM/DSS or AOM only was determined by Mann-Whitney U tests (**p ≤ 0.01). **(C)** Size distribution of lesions in colons of B6 and A/J mice. Data is presented as median number of lesions in each lesion size category for each group, plotted by treatment. The smallest size category includes lesions with 1–4 aberrant crypts, while lesions are considered tumors if they contain ≥30 abnormal crypts per lesion (0.4 mm^2^). **(D)** Number, load and average size of small intestinal lesions in B6 or A/J mice treated with AOM/DSS (++) or AOM only (+). Box plots show median (line), mean (+), IQR (box) and minimum to maximum (whiskers)

ACFs were detected in all AOM/DSS– and AOM–treated animals. Following AOM/DSS treatment, the total number, load and average size of the ACFs were similar in both B6 and A/J strains, indicating a comparable development of these preneoplastic lesions (Fig. 3A). However, the multiplicity of larger lesions categorized as tumors was significantly higher in A/J mice compared to B6 (*p* = 0.008). Tumors were detected in all AOM/DSS–treated mice, and the counts ranged from 2 to 19 in B6 to 30–56 in A/J. The average size of the tumors was not significantly different between the two strains (*p* = 0.548), while tumor load was significantly higher in the A/J (*p* = 0.008), indicating the load was influenced by several tumors rather than a few very large (Fig. 3B). The size distribution of lesions illustrates that AOM with DSS promotion led to development of a markedly higher number of larger lesions in the A/J mice compared to B6 (Fig. 3C).

Following AOM treatment without DSS promotion, all animals of both strains developed ACFs, indicating that AOM injection alone can induce carcinogenesis in both strains. The A/J mice did, however, develop significantly higher abundance, size and load of ACFs compared to B6 (Fig. 3A). All AOM-treated A/J mice developed tumors, while no B6 mice treated with only AOM developed lesions categorized as tumors. Tumor count ranged between 1 and 20 for the AOM–treated A/J mice (Fig. 3B, C). These findings indicate that AOM without DSS promotion effectively led to development of ACFs as well as tumors in A/J mice, while this treatment was ineffective in B6 mice.

Min mice on both B6 and A/J background have been shown to also develop small intestinal lesions [36, 37]. Thus, the small intestines (SI) for all individuals were also examined and scored. For SI lesions, average counts were < 5 for both strains (Fig. 3D). Neither in the AOM/DSS-treated nor the AOM-treated groups were there significant differences in number, average size or lesion load detected between the two strains.

### Histopathological characterization of colonic lesions

**Fig. 4:**
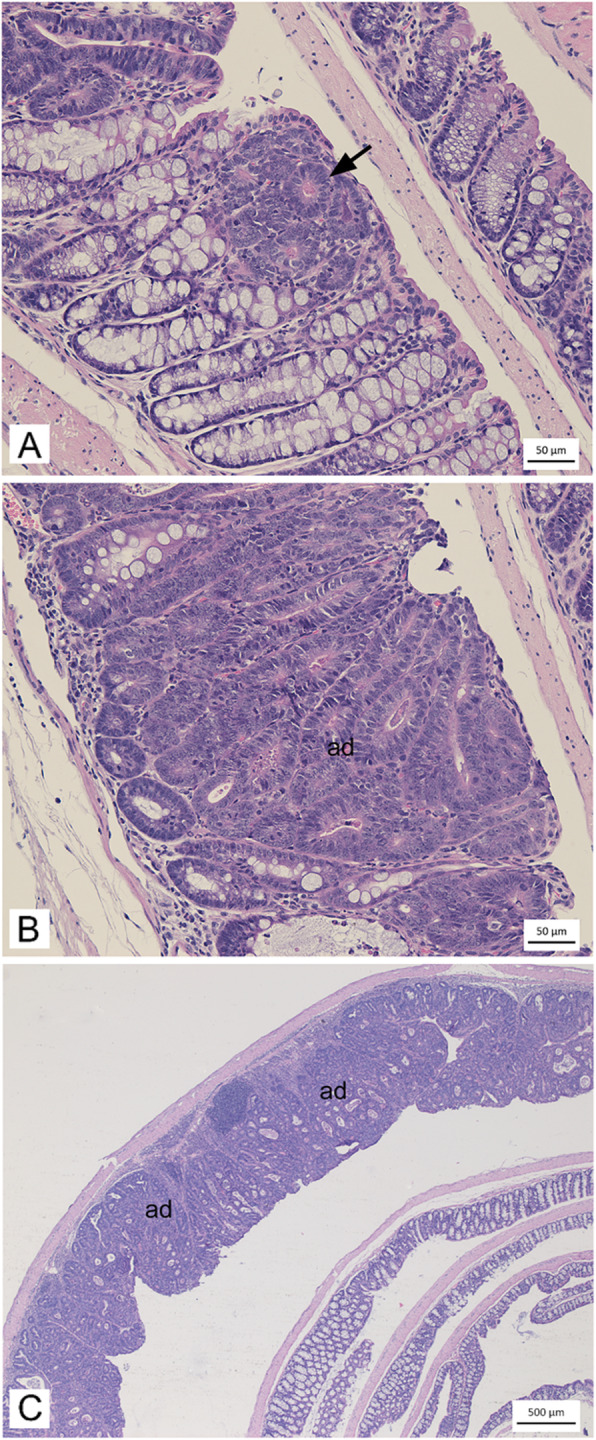
Examples of morphological features of colonic lesions. HE-stained Swiss roll sections showing **(A)** a small area with crypt cell hyperplasia (arrow), **(B)** a moderately sized adenoma (ad) located in the lamina propria, and **(C)** large, continuous adenoma (ad) present in the mucosa. The presented pictures were taken of colons from one AOM-treated A/J mouse (A, B) and one AOM/DSS-treated A/J mouse (C)

To further characterize the lesions, histopathological evaluation of colons from the two AOM/DSS–treated groups was conducted (Table 1). As a relatively high number of colonic lesions was observed in A/J mice treated with AOM only, this group was also included for histopathological assessment. Colonic lesions were classified as hyperplasia/dysplasia, adenomas or carcinomas according to morphological features as visualized in Fig. 4. Hyperplasia/dysplasia was detected in all AOM/DSS–treated mice independent of strain. For the AOM/DSS–treated animals, adenomas were recorded in all A/J mice, and in 3 out of 5 B6 mice. No carcinomas were detected in any of the groups assessed. The count of both hyperplasia/dysplasia and adenomas were significantly higher in the A/J mice compared to B6 mice (both *p* = 0.008). Moreover, the size of adenomas, as shown in Fig. 4, was larger in the AOM/DSS–treated A/J mice than the AOM/DSS–treated B6 mice. For the AOM–treated A/J mice, hyperplasia/dysplasia was detected in 2 out of 5 and adenomas in only 1 out of 5 (Table 1).

**Table 1:** Histopathological classification of colonic lesions

	AOM/DSS (++)				AOM (+)	
	B6		A/J		A/J	
**Hyperplasia/dysplasia**	5/5	2.20 ± 1.64	5/5	9.80 ± 1.79	2/5	1.80 ± 3.49
**Adenoma**	3/5	1.00 ± 1.00	5/5	14.20 ± 2.86	1/5	1.20 ± 2.68
**Carcinoma**	0/5	0.00 ± 0.00	0/5	0.00 ± 0.00	0/5	0.00 ± 0.00

Colonic lesions from B6 or A/J mice were classified as hyperplasia/dysplasia, adenomas or carcinomas. Data shows the number of individual mice in which at least one lesion within the class was detected, and mean numbers of lesions detected within each class (mean ± SD), in the given experimental groups.

## Discussion

Since the first reports of the AOM/DSS model for induction of colorectal carcinogenesis, several variations of its implementation have been employed. Commonly used AOM/DSS models employing high doses of DSS are likely to inflict advanced disease and detrimental effects in the animals, which for many study purposes may be avoidable. For studies aiming to detect possible risk factors for, modifiers of, or chemopreventive agents that arrest the carcinogenesis, it may be advantageous to assess the early phases of CRC. To date, the majority of studies using the AOM/DSS model have employed DSS doses of 2% or higher [14, 17, 22, 38, 39], although previous reports have shown clear tumor-promoting effects of lower doses. Male ICR [24] and B6 mice [25] subjected to a single AOM injection (10 mg/kg, i.p) followed by a 7- or 4-day administration of 1% in drinking water showed consistent tumor development within 14 to 18 weeks after injection, respectively. A substantial number of mice in these two studies developed both adenomas and adenocarcinomas, possible due to the duration of the trials. In ICR mice, a protocol of a single i.p. injection of AOM followed by a 7-day administration of 1% DSS yielded adenomas and adenocarcinomas in all mice after 20 weeks [28].

Shorter-term experiments in which preneoplastic lesions is used as a biomarker has been advised to minimize animal suffering in CRC experiments [34]. We show that by using an AOM/DSS model involving a single AOM injection (10 mg/kg, s.c.) followed by repeated administration (3 × 7 days) of a low dose (1%) DSS, we could consistently detect ACFs as well as hyperplasia/dysplasia in both B6 and A/J mice after 12 weeks. These findings indicate that a dosage of 1% DSS can be sufficient in the AOM/DSS model for study of early phase carcinogenesis, when using preneoplastic lesions as the outcome measure. Adenomas were detected in the majority of B6 mice and in all A/J mice subjected to our modified protocol, highlighting differential strain susceptibility to the AOM/DSS model as further discussed below. An important note is that ACFs can be distinguished into classical and flat ACFs, where classical ACFs are elevated from the mucosa. Although both flat and classical ACFs have been detected in AOM-treated A/J mice previously, flat ACFs are reportedly most likely to develop into tumors [40]. In the current study, we could not elucidate if a higher proportion of classical ACFs rather than flat ACFs were developed in B6 compared to A/J, and whether this played a role for the progression of lesions into adenomas.

We sought to carefully assess clinical symptoms during the trial. We detected blood in feces in AOM/DSS–treated mice despite the low dose of DSS, and thus clinical symptoms were not avoided with our approach. The symptoms accompanied disease progression, as emphasized by more prevalent symptoms in the A/J mice which consistently developed adenomas and larger lesions compared to B6. A recent report on methods to assess affective state in AOM/DSS–treated B6 mice has provided extensive data on clinical symptoms throughout the trial [41]. In the study by Chartier et al., the AOM/DSS protocol involved a single injection of AOM (7.4 mg/g, i.p.) followed by three cycles of 7-day 2% DSS treatment. Although divergent from our employed protocol, the data from this previous study showed that rectal bleeding occurred at the first cycle of DSS treatment, and symptoms such as under-condition and bodyweight loss were either sustained or progressed along the trial. Early symptoms including blood in feces have been shown to manifest immediately upon high-dose DSS treatment in B6 mice [20]. In our study, symptoms manifested during the 2nd cycle of DSS treatment in both strains, indicating the animal suffering can be alleviated by reduction of DSS concentration. The symptoms did not persist in-between treatment cycles, demonstrating recovery among the animals. Taken together, reducing the dose of DSS could contribute to reduced risk of early trial terminations in due to reached humane endpoints in studies employing the AOM/DSS model.

In addition to effective induction of carcinogenesis with the combinatory AOM and DSS treatment, we found that a single AOM injection induced ACF formation in A/J mice, supportive of previous studies [40, 42, 43]. On the contrary, we detected no ACFs in response to exposure of AOM alone in B6 mice, demonstrating a promotion using DSS is necessary to induce carcinogenesis in this strain. This is in agreement with previous studies in which no colonic tumors were detected in B6 mice given only AOM or only 1% DSS [25], and that A/J mice are less susceptible than B6 mice to inflammation induced by 3% DSS, yet more susceptible to the combined AOM/DSS-induced CRC [44]. Studies have investigated strain variances with respect to genetics [44, 45], gut microbiota and mucosal immune system [46–48], inflammatory response [49] and CYP-dependent metabolism [50] that may all contribute to the differential susceptibility observed in our study as well as in previous reports.

In general, age and gender have been reported to influence the susceptibility of mice to AOM and/or DSS. Males have been shown to develop more adenomas compared to females [51] and AOM injection at a younger age increase the tumorigenic response [52, 53]. Beyond mouse strain, DSS concentrations and frequency of administration, responses to DSS are highly affected by housing facility, molecular weight of DSS, and mouse microbiota composition [18]. Gut microbiota in laboratory mice vary across facilities, and has been shown to alter the outcomes in several disease models [54, 55]. Thus, pilot testing of the present experimental setup is encouraged for each individual laboratory, in accord with good practice when introducing new protocols.

## Conclusions

In summary, we show that an AOM/DSS model with a low dose of DSS can be used to reliably induce colorectal carcinogenesis measured as preneoplastic lesions in both B6 and A/J mouse strains while limiting severe symptoms. This study highlights the importance of adjusting the treatment regimen according to mouse strain and study purposes in futures studies employing the AOM/DSS model.

## Methods

### Ethical considerations

The experiment was approved by the Norwegian Animal Research Authority (FOTS ID 15446). The study was conducted at the Section for Experimental Biomedicine, Faculty of Veterinary Medicine, NMBU, Oslo, in accordance with local and national regulations for laboratory animal experiments. The animal facility is licensed by the Norwegian Food Safety Authority, and the health of the animals were monitored following a program recommended by the Federation of European Laboratory Animal Science Association (FELASA).

### Animals and husbandry

Wild-type A/J mice were bred at the Department of Experimental Biomedicine at NMBU, Norway as previously described [36]. A total of 15 purchased female inbred C57BL/6JRj (B6; Janvier Labs, Saint-Berthevin Cedex, France) mice aged 7 weeks, and 15 female inbred A/J wild-type mice aged 7–9 weeks were randomized to six groups based on strain. The groups were administered either NaCl injection combined with distilled H_2_O administration (control treatment), AOM injection, or AOM injection combined with DSS treatment (Fig. 1). The mice were maintained in closed type III individually ventilated cages (Allentown Inc., USA) under standard conditions (12 h light/dark cycle, 21 ± 2 °C, 20 air changes per hour, and 45 ± 5% relative humidity). All cages contained standard aspen bedding, cellulose nesting material and red polycarbonate houses (Tecniplast, Buguggiate, Italy). Tap water and standard chow diet (RM1(E), SDS; Special Diet Services, Witham, United Kingdom) was provided ad libitum. The cages, bedding, nesting material and water bottles were changed minimum once a week.

### Experimental procedure and health monitoring

A 10 mg/mL stock solution of AOM in sterile H_2_O was prepared from 25 mg AOM (Sigma-Aldrich, #A5486). Fresh 1 mg/mL working solutions of AOM was prepared by addition of sterile NaCl (0.9%, B.Braun) prior to use. The animals were injected subcutaneously (s.c.) into the neck fold with 10 mg/kg AOM working solution, while animals were under transient anesthesia (sevofluorane, 3%, 200 mL/min). The control treatment entailed one single s.c. injection of sterile NaCl solution (10 mg/kg). Injection volume was rounded to nearest 10 μL. For DSS administration, DSS (36,000–50,000 M.Wt., Colitis Grade, MP Biomedicals) was dissolved in distilled H_2_O (1%, w/v) just prior to supply. Control treatment entailed the same regimen with fresh distilled H_2_O only. DSS or distilled H_2_O was administered in three 7-day cycles (day 7–14, 32–39, 56–63). Fresh DSS solution as well as distilled H_2_O was prepared and supplied every 2nd day throughout the 7-day cycles. For 16 days between the 1st and 2nd cycles, and 18 days between the 2nd and 3rd cycle, the animals were given regular tap water and allowed to recover. Animals were euthanized 18 days after the last cycle of DSS/control treatment.

Health monitoring was performed daily for all animals. For the AOM/DSS–treated animals, welfare was recorded every 2nd day during the DSS/control treatment regimen by use of a customized score sheet for bodyweight, appearance and behavior, rectal bleeding and rectal prolapse (**S1 dataset**). Assessment of appearance and behavior included evaluation of overall condition, activity, movement and facial expression of pain. Blood in feces was recorded as rectal bleeding. Animals exhibiting any symptom was kept under close observation. Humane endpoints were defined as follows; body weight loss > 15%, rectal bleeding defined as blood around anus sustained over two subsequent days, a complete bulging of distal colon out of rectum, and severely under-conditioned appearance and behavior.

### Scoring of intestinal lesions

Colons and small intestines were harvested and briefly flushed with PBS. For scoring of intestinal lesions, colons were prepared as described previously [56]. Briefly, each intestinal segment was fixated flat between two filter papers in formalin solution (10%, neutral buffered; VWR Chemicals) for 24 h prior to staining with methylene blue (MB) solution (Sigma-Aldrich; 0.1% in 10% neutral buffered formalin). The intestinal segments were stored refrigerated in 70% ethanol until analysis. The identification of intestinal lesions was performed by microscopy according to previously described procedure [57]. An inverted light microscope (CKX41, Olympus Inc., Hamburg, Germany) equipped with a digital color camera (DP25, Olympus Inc., Hamburg, Germany) was used to examine the colons for lesions.

Colonic lesions were classified in two categories; aberrant crypt foci (ACF) and tumor. ACFs can be recognized and distinguished from normal epithelia based on MB staining [8–10, 58]. In the current study, the lesions were not further distinguished. Diameters of lesions were measured using an eye piece graticule, and colonic lesion size (mm^2^) was calculated based on the measured diameters. All recorded lesions were grouped into lesion size classes (0.002–0.008 mm^2^, 0.009–0.064 mm^2^, 0.065–0.512 mm^2^, 0.513–4.096 mm^2^ and ≥ 4.097 mm^2^) as previously defined [36, 56]. Lesions were considered tumors if they contained ≥30 abnormal crypts per lesion (0.4 mm^2^). The total number of lesions, lesion load and distribution were measured and calculated per mouse in order to study lesion development in the intestines. Lesion load was defined as the sum of the area of all lesions (ACFs or tumors) observed in an intestine. For the small intestines, ACFs are not present and thus only lesions were recorded. Scoring of intestinal lesions are rendered in supplementary material (**S2 dataset**).

### Histopathology

Histopathological classification of colonic lesions was conducted on colons from all AOM/DSS–treated individuals and AOM-treated A/J mice. Following the scoring of lesions, swiss rolls were made of the colons, using a procedure first described by Moolenbeek and Ruitenberg [59] and further modified by Sodring et al. [36]. Briefly, colons were rolled lengthwise from proximal to distal, with the mucosa facing inwards, and embedded in paraffin blocks. For each paraffin-embedded colon, sections (3 μm thick) were made at three different depths (top, middle, bottom). The sections were stained with hematoxylin and eosin (HE) and examined blindly by a pathologist using a light microscope. Lesions were classified as hyperplasia/dysplasia, adenomas (tumors restricted to the mucosa) or carcinomas (tumors with distinct infiltrative growth through the mucosa into the submucosa).

### Statistical analyses

Statistical analyses were run in JMP® Pro 15 (SAS, NC, USA) or GraphPad Prism (v6.07 and v9.1.1; GraphPad Software, Inc., La Jolla, USA). Figures were created using GraphPad Prism and Inkscape (v0.92.4; http://www.inkscape.org/). Applied statistical methods are specified in figure legends. Normality was controlled by Normal Quantile plot and D’Agostino-Pearson test. For non-normally distributed variables, including the ordinal data, Mann-Whitney U tests were conducted on non-transformed data.

## Supplementary Information

**Additional file 1 S1 Dataset**. Records of bodyweight, health monitoring and DSS consumption (XLSX).

**Additional file 2 S2 Dataset**. Identification and classification of intestinal lesions (XLSX).

## Data Availability

The datasets used and/or analyzed in this study is available within the provided supplementary material.

## Abbreviations

ACF: Aberrant crypt foci
AOM: Azoxymethane
B6: C57BL/6J
CRC: Colorectal cancer
DSS: Dextran sulfate sodium
